# GxcDD, a putative RacGEF, is involved in *Dictyostelium *development

**DOI:** 10.1186/1471-2121-8-23

**Published:** 2007-06-20

**Authors:** Subhanjan Mondal, Dhamodharan Neelamegan, Francisco Rivero, Angelika A Noegel

**Affiliations:** 1 Institute for Biochemistry I, Medical Faculty, and Center for Molecular Medicine, University of Cologne, 50931, Cologne, Germany; 2 National Research Council, Institute for Biological Sciences, 100 Sussex Drive, Ottawa ON, Canada

## Abstract

**Background:**

Rho subfamily GTPases are implicated in a large number of actin-related processes. They shuttle from an inactive GDP-bound form to an active GTP-bound form. This reaction is catalysed by Guanine nucleotide exchange factor (GEFs). GTPase activating proteins (GAPs) help the GTPase return to the inactive GDP-bound form. The social amoeba *Dictyostelium discoideum *lacks a Rho or Cdc42 ortholog but has several Rac related GTPases. Compared to our understanding of the downstream effects of Racs our understanding of upstream mechanisms that activate Rac GTPases is relatively poor.

**Results:**

We report on GxcDD (**G**uanine e**x**change factor for Ra**c **GTPases), a *Dictyostelium *RacGEF. GxcDD is a 180-kDa multidomain protein containing a type 3 CH domain, two IQ motifs, three PH domains, a RhoGEF domain and an ArfGAP domain. Inactivation of the gene results in defective streaming during development under different conditions and a delay in developmental timing. The characterization of single domains revealed that the CH domain of GxcDD functions as a membrane association domain, the RhoGEF domain can physically interact with a subset of Rac GTPases, and the ArfGAP-PH tandem accumulates in cortical regions of the cell and on phagosomes. Our results also suggest that a conformational change may be required for activation of GxcDD, which would be important for its downstream signaling.

**Conclusion:**

The data indicate that GxcDD is involved in proper streaming and development. We propose that GxcDD is not only a component of the Rac signaling pathway in *Dictyostelium*, but is also involved in integrating different signals. We provide evidence for a Calponin Homology domain acting as a membrane association domain. GxcDD can bind to several Rac GTPases, but its function as a nucleotide exchange factor needs to be studied further.

## Background

Rho GTPases are small monomeric GTPases of the Ras superfamily. Like any other GTPase Rho GTPases act as binary molecular switches cycling between a GTP-bound active and a GDP-bound inactive form. Guanine nucleotide exchange factors (GEFs) catalyze the activation reaction, and GTPase activating proteins (GAPs) convert the active to an inactive form. Further regulators, guanine nucleotide dissociation inhibitors (GDIs), block spontaneous activation and regulate cycling between membrane and cytosol. When activated, Rho GTPases undergo a conformational change enabling them to interact with their effector molecules and transduce signals for downstream events. Rho GTPases have been implicated in a large number of actin-related processes like motility, adhesion, morphogenesis, membrane trafficking and cytokinesis [1,2].

The human genome codes for 21 Rho GTPases and functions of most of them are only poorly understood. Of these, three, namely RhoA, Rac1 and Cdc42 are more extensively studied. RhoA generates myosin-based contractility and formation of adhesion complexes; Rac1 and Cdc42 are primarily involved in formation of protrusive structures, Rac1 regulates formation of lamellipodia and Cdc42 regulates filopodia formation and establishment of cell polarity [1,2].

Sequencing of the genome of the social amoeba *Dictyostelium discoideum *revealed the presence of 18 Rac related GTPases, whereas a typical Rho or Cdc42 were absent  [3,4]. Only a few of the Rac related GTPases have been characterized in detail. Rac1A, 1B and 1C [5,6] and Rac E are required for cytokinesis [7], Rac1A was also shown to be involved in a formin-dependent pathway for filopodia formation [8], RacB is required for chemotaxis and morphogenesis [9] and RacC has been implicated in phagocytosis [10] and plays an important role in PI 3-kinase activation and WASP activation for the dynamic regulation of F-actin assembly during chemotaxis [11]. RacG is required for cell shape, motility, and phagocytosis [12] and RacH has been implicated in vesicular trafficking [13].

Compared to our understanding of the downstream effects of Rac GTPases less is known about the mechanisms that activate Rac GTPases controlled by GEFs, GAPs or GDIs. In *Dictyostelium *at least 45 proteins contain a RhoGEF-PH module and most of them have a unique domain composition. The RhoGEF-PH (or diffuse B-cell lymphoma homology DH/pleckstrin homology PH) module is the structural feature that mediates the nucleotide exchange activity on Rho GTPases. Five of these RacGEFs have been studied in some detail. DdRacGap1 (DRG) containing both RhoGEF and Rho-GAP domains acts as a GEF for Rac1 and simultaneously acts as a GAP for RacE and Rab GTPases [14]. RacGEF1 has a specificity for RacB in regulating chemoattractant stimulation, F-actin polymerization, and chemotaxis [9]. The tail domain of MyoM, an unconventional myosin has been shown to catalyse nucleotide exchange on Rac1 GTPases and can induce actin-driven surface protrusions [15]. More recently Trix, a three CH domain containing RacGEF, has been suggested to regulate the endocytic pathway [16]. Finally, Darlin, an armadillo repeat protein homologous to the mammalian GEF smgGDS (small G-protein dissociation stimulator, a guanine nucleotide exchange factor for numerous Ras and Rho family GTPases [17]), has been shown to physically interact with RacE and RacC and may modulate chemotactic responses during early development [18]. Nucleotide exchange activity on Rho GTPases are also displayed by CZH (CDM-zizimin homology) domain proteins [19]. Presently only a few members of the family have been studied in other organisms like the mammalian Dock180 and CED-5 in *C. elegans*. *Dictyostelium *has 8 members of this family, but the functions of them remain to be elucidated.

In this study we focus on GxcDD, a novel multidomain RacGEF that contains a calponin homology (CH) domain, two IQ motifs, a DH domain, three PH domains and an ArfGAP domain. We show that, though dispensable for growth and development, GxcDD is required for proper streaming early in *Dictyostelium *development. The RacGEF domain of GxcDD can physically interact with several Rac  GTPases. Characterization of the individual domains revealed that the CH domain can act as a membrane anchor and the ArfGAP-PH tandem accumulates at cortical regions and on phagosomes. Our data also suggest that a conformational change is possibly required to activate GxcDD.

## Results

### Expression pattern and domain characterization of GxcDD

**Figure 1:**
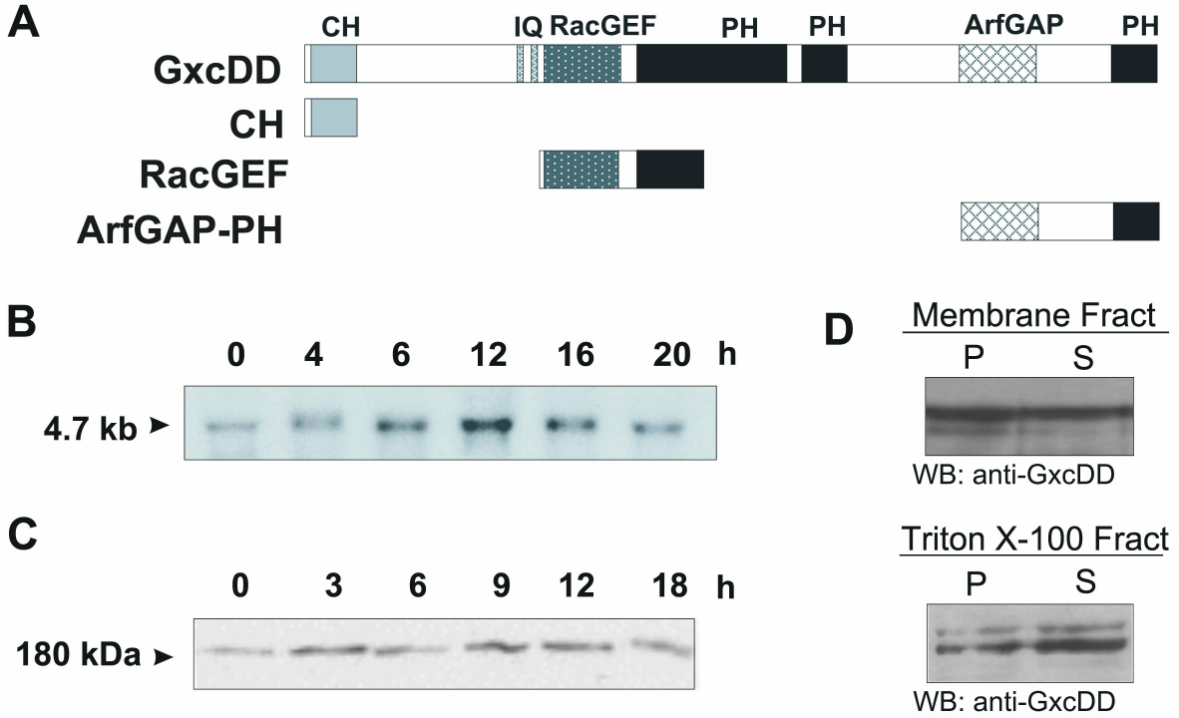
Architecture, expression profile and subcellular localization of GxcDD. (A) Schematic representation of the domain organization of GxcDD and the domain constructs used in the study. (B) Northern blot showing the expression profile of GxcDD during *Dictyostelium *development on phosphate agar plates. Cells were harvested at the indicated time points and RNA isolated by phenol-chloroform and probed using a fragment derived from the 3' end of the GxcDD cDNA (nt 3807–4860). (C) Western blot showing the accumulation of GxcDD during development. Total cellular proteins were harvested at the indicated time points and subjected to western blot analysis using polyclonal antibodies raised against GxcDD. (D) (top) Membrane (P) or cytosolic fractions (S) and (bottom) Triton X-100 soluble (S) and insoluble (P) cytoskeletal fractions were prepared as described in Materials and methods and subjected to immunodetection using GxcDD polyclonal antibodies.

The gene coding for the RacGEF GxcDD (**G**uanine e**X**change factor for ra**C**) is located on chromosome 3. GxcDD is 1619 residues long with a calculated molecular mass of 179.652. It is a multidomain protein containing a CH domain, two IQ motifs, three PH domains, a RhoGEF domain and an ArfGAP domain (Fig. 1A). The calponin homology (CH) domain is located at the N terminus and resides between residues 18–122, the two IQ motifs between residues 390–417 and 432–461. The DH domain of RhoGEFs is the region required for mediating guanine nucleotide exchange on the Rho family GTPases. In general, a pleckstrin homology (PH) domain follows the DH (*d*iffuse B-cell lymphoma *h*omology) domain and this tandem DH-PH module is the signature motif of the Dbl family of guanine nucleotide exchange factors (GEFs). Similarly, in GxcDD, a DH domain responsible for the catalytic RacGEF activity resides between residues 464–637. There are three PH domains in GxcDD, a comparatively large and less defined PH domain between residues 664–910 and two more between residues 941–1038 and 1520–1618. An ArfGAP domain, which shows highest homology to centaurin-α is placed between residues 1258–1376. Centaurins are ArfGAPs with functions in intracellular trafficking and contain a PH domain. Centaurins act downstream of PI 3 kinases and are targets for PtdIns(3,4,5)P_3 _[20].

We analysed the expression profile of GxcDD during *Dictyostelium *development at the transcript level with a specific cDNA probe and at the protein level with polyclonal antibodies, respectively. We found that GxcDD is expressed throughout development and that the protein levels do not vary greatly (Fig. 1B, C). As the polyclonal antibodies that had been raised against the ArfGAP-PH domain of GxcDD, were unsuitable for immunofluorescence, we addressed the subcellular localization of the protein by means of subcellular fractionation and Triton X-100 treatment of the cells. We found GxcDD to be equally present in the cytosolic and membranous fractions. It also associated with the Triton X-100 insoluble cytoskeletal fraction (Fig. 1D).

### The CH domain of GxcDD functions as a membrane association domain

**Figure 2:**
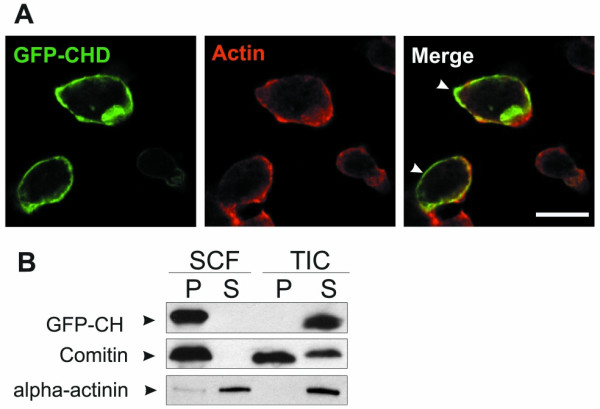
The CH domain of GxcDD acts as a membrane anchor. (A) Cells expressing GFP-CH were fixed and stained with actin specific mAb act1-7. GFP-CH and actin co-localized only partially as indicated by arrows. (B) Subcellular fractionation (SCF) and Triton X-100 insoluble fractionations (TIC) of cells expressing GFP-CH were done as described in Materials and methods. Supernatant (S) and pellet (P) fractions were analysed using a GFP monoclonal antibody. Comitin is used as a marker for the membrane fraction and α-actinin as a marker for the cytosolic fraction. GFP-CH was exclusively found in the membrane fractions and did not associate with the cytoskeleton.

To study the function of the CH domain in GxcDD we expressed it as a fusion with GFP in *D. discoideum*. Live cell imaging of GFP-CH showed that it almost completely localized to the cortical regions of the cell, suggesting it may either be associated with the cortical actin cytoskeleton or the plasma membrane. When GFP-CH expressing cells were stained for actin, we observed only a partial overlap at certain places, which might be due to the close proximity of cortical actin and the plasma membrane (Fig. 2A). Furthermore, subcellular fractionation revealed that GFP-CH was completely recovered in the membrane fraction (Fig 2B, lanes 1 and 2). When Triton-insoluble cytoskeletons were prepared we found GFP-CH exclusively in the supernatant fraction indicating that it does not associate with actin (Fig. 2B, lanes 3 and 4). For control we used comitin, a membrane and actin cytoskeleton associated protein, and α-actinin, an F-actin crosslinking protein. Comitin is present in the membrane fraction and also in the cytoskeletal fraction, whereas α-actinin does not associate with the Triton-insoluble cytoskeleton due to its low affinity for F-actin. These findings demonstrate that the CH domain of GxcDD does not interact with actin but, surprisingly, associates with membranes. The CH domain of GxcDD thus can act as a membrane anchor for GxcDD.

### Association of GxcDD with *Dictyostelium *Rac GTPases

**Figure 3:**
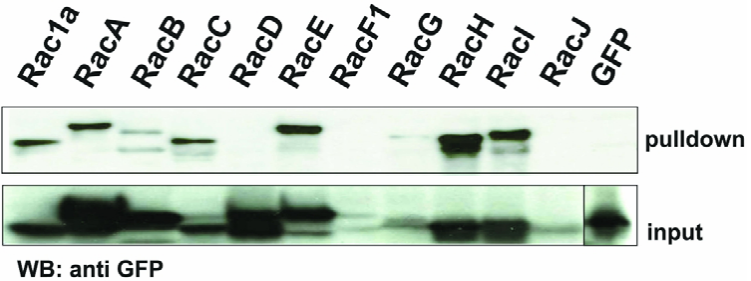
Association of GxcDD with *Dictyostelium *Rac GTPases. GST-RacGEF was expressed in *E. coli *and bound to glutathione-sepharose beads. Equal amounts of washed beads were incubated with cell lysates of AX2 cells expressing GFP fusions of 11 of 18 *Dictyostelium *Racs. Pulldown eluates were resolved by 12% SDS-PAGE and the proteins detected using a GFP monoclonal antibody.

As the *Dictyostelium *genome lacks typical Rho or Cdc42 small GTPases, but has several Rac related GTPases we checked for a direct physical interaction of the DH domain of GxcDD with *Dictyostelium *Rac GTPases. We expressed the DH domain of GxcDD as a GST fusion protein (GST-DH) in *E. coli*, bound the protein to glutathione-sepharose beads and used them for incubation with lysates derived from *Dictyostelium *cells expressing different Rac GTPases as GFP fusion proteins. Rac GTPases interacting with GST-DH were identified by immunoblotting with GFP monoclonal antibodies (Fig. 3). The expression levels of the Rac proteins varied, and RacF1, G and J were not expressed in very high amounts, whereas Rac1a, A, B, C, D, E, H and I were highly expressed. We found that Rac1a, A, C, E, H and I bound to the DH domain of GxcDD, whereas RacB and RacD, that were expressed in very high amounts, did not bind to the DH domain, indicating specificity in the pulldown assay. The data indicate that GxcDD can activate more than one Rac. It is however also likely that it is not the only exchange factor for these interacting proteins.

### The C terminal domain of GxcDD is enriched in the cortex and relocates to the membrane during phagocytosis

**Figure 4:**
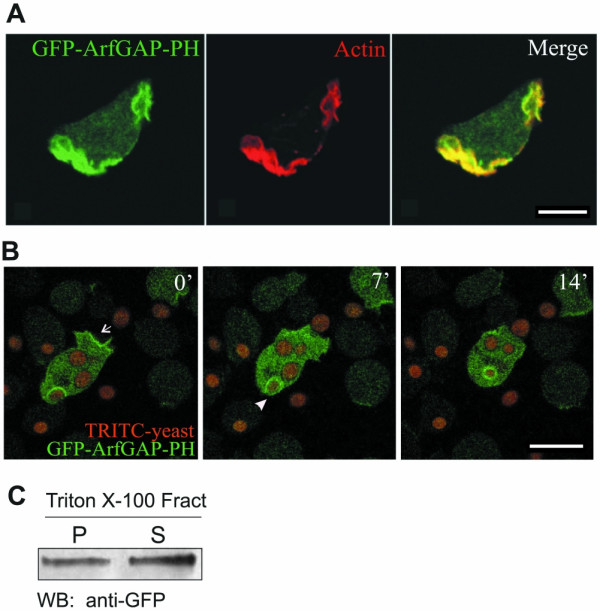
Cortical localization of the C-terminus of GxcDD containing the ArfGAP-PH tandem and enrichment on phagosomes. (A) Wild type *Dictyostelium *cells expressing GFP-ArfGAP-PH were fixed and stained with actin specific mAb act1-7, showing complete overlap of the two. (B) Live cell imaging of cells expressing GFP-ArfGAP-PH incubated with TRITC labelled yeast. GFP-ArfGAP-PH enriches at the phagocytic cup (arrow head) and also at the leading edges of the cell (arrow). Pictures captured at different times are shown. (C) Cells expressing GFP-ArfGAP-PH were subjected to Triton X-100 extraction. Supernatant (S) and pellet (P) fractions were separated by 10% SDS-PAGE and the protein immunodetected using GFP specific mAb K3-184-2. GFP-ArfGAP-PH was equally distributed in both the Triton X-100 insoluble cytoskeleton and the soluble fraction.

At the C terminus GxcDD possesses an ArfGAP domain followed by a PH domain. Arfs are small GTPases known to be required for vesicular trafficking. An ArfGAP domain would be required to inactivate the activated form of Arf [21]. Similar to the association of PH domains with DH domains, the ArfGAP domain in GxcDD is associated with a PH domain. To examine the functions of these domains we expressed the C-terminal part containing the ArfGAP-PH tandem as a GFP fusion protein in AX2 cells. GFP-ArfGAP-PH was targeted to the cell cortex where it co-localized with actin (Fig. 4A). Live-cell imaging experiments where GFP-ArfGAP-PH expressing cells were incubated with TRITC labelled yeast particles showed a strong enrichment of the fusion protein on phagosomes and at the leading edges of the cell (Fig. 4B). Cell fractionation using Triton X-100 indicated a distribution of GFP-ArfGAP-PH in both the Triton-insoluble cytoskeleton fraction and in the supernatant (Fig. 4C). These results suggest that unlike the N-terminal CH domain, which is totally membrane bound, the C-terminal part can also associate with the actin cytoskeleton.

**Figure 5:**
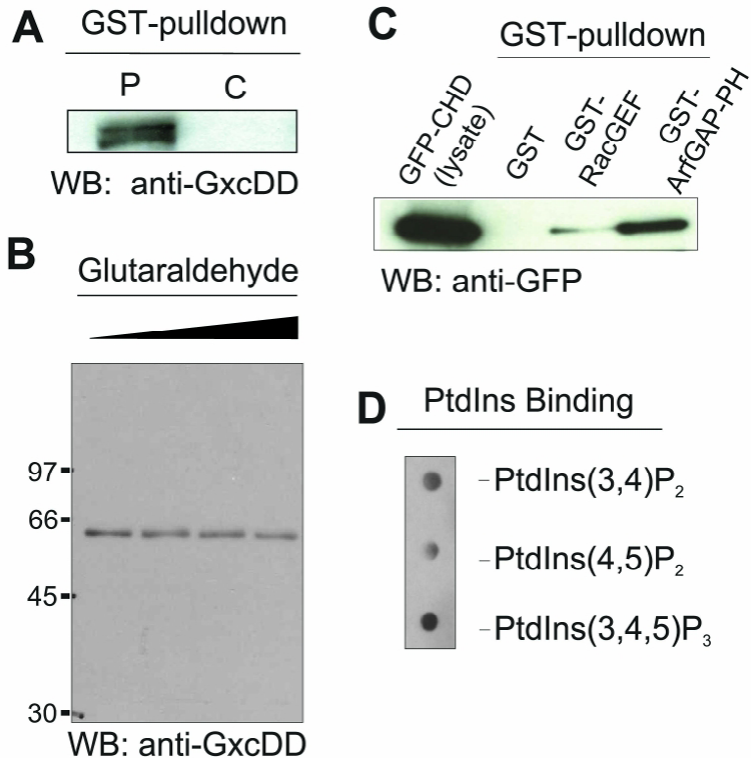
Interaction of ArfGAP-PH with full length GxcDD and phosphoinositides. (A) GST-ArfGAP-PH was expressed in *E. coli *and bound to glutathione-sepharose beads. Beads were incubated with wild type cell lysates (P). GST bound beads were used as a control (C). Pulldown eluates were resolved by 10% SDS-PAGE, western blots revealed GxcDD as an interacting protein. (B) GST-ArfGAP-PH was cleaved by thrombin to liberate the ArfGAP-PH protein and purified protein was tested for oligomerization using increasing amounts of glutaraldehyde as a crosslinking agent (0 – 0.1% v/v). (C) The CH domain has the potential to bind to the GEF domain and the ArfGAP-PH domain. Lysates from cells expressing GFP-CH domain (GFP-CHD) were used in pull down assays employing GST-RacGEF or GST-ArfGAP-PH bound to glutathione-sepharose beads. The blot was probed with a GFP-specific monoclonal antibody. (D) PtdIns(3,5)P_2_, PtdIns(4,5)P_2_, PtdIns(3,4,5)P_3 _were spotted on a PVDF membrane and incubated with ArfGAP-PH protein and binding detected using GxcDD specific polyclonal antibodies.

When we used GST-ArfGAP-PH to identify interacting proteins we could co-precipitate full length GxcDD from AX2 cell lysates. The precipitated proteins were resolved on SDS polyacrylamide gels and one of the unique bands analysed by MALDI-TOF mass spectroscopy identified GxcDD as an interacting protein. Co-precipitation of GxcDD was also confirmed by western blot analysis (Fig. 5A). To test whether the ArfGAP-PH domain interacts with itself and forms higher oligomers, we purified the ArfGAP-PH domain by thrombin cleavage from the GST fusion protein and treated it with increasing amounts of glutaraldehyde to promote crosslinking of associated proteins. The proteins were resolved on SDS polyacrylamide gels, blotted and probed with the GxcDD specific polyclonal antibodies. We did not observe formation of higher oligomers (Fig. 5B), indicating that the association of ArfGAP-PH with the full length GxcDD is through an interaction with other domains in the protein. We then checked if the N-terminal CH domain could interact with either the RacGEF domain or the ArfGAP-PH tandem in a GST-pulldown experiment using cells expressing the GFP-CH domain. We found that beads coated with ArfGAP-PH tandem could pulldown significant amounts of GFP-CH domain, as compared to beads coated with the RacGEF domain. Thus the CH domain at the N terminus can be an interacting domain for the ArfGAP-PH tandem and this interaction may regulate either the activity or localization of the protein.

PH domains bind to phosphatidylinositol phosphates (PtdIns) and mediate the recruitment of proteins to membranes. When we tested whether the PH domain in the ArfGAP-PH domain could bind to PtdIns using a dot blot assay we found that the protein bound to PtdIns(3,4)P_2 _and PtdIns(4,5)P_2_, but highest binding was observed with PtdIns(3,4,5)P_3_, the product of PI3K (Fig. 5D). The PH-domain associated with the RacGEF domain could not be tested for binding to phosphatidylinositol phosphates so far as the protein was insoluble when expressed in *E. coli*.

### Characterization of *gxcDD*^- ^cells

**Figure 6:**
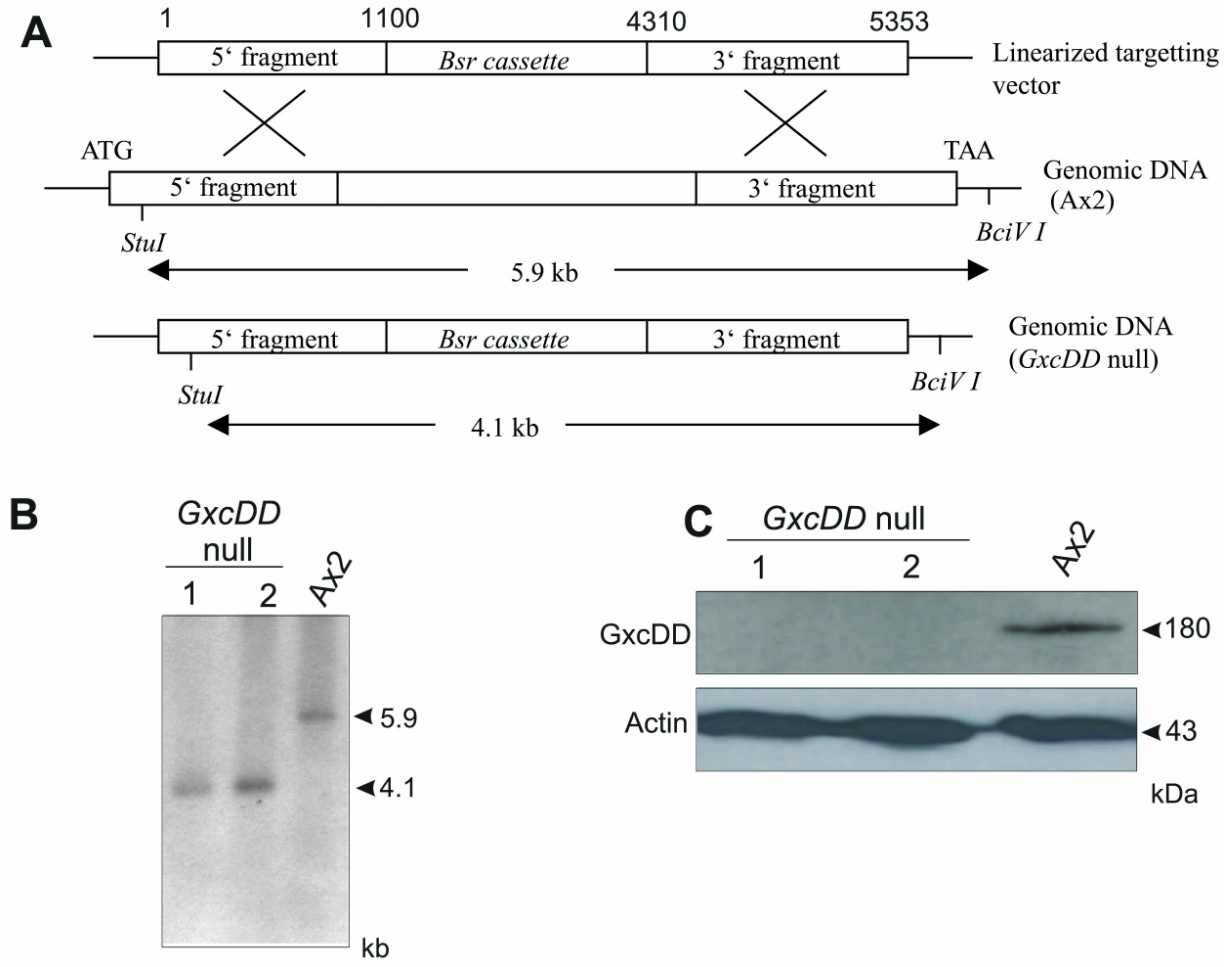
Generation of *gxcDD*^- ^cells. (A) Schematic representation of the strategy to generate *gxcDD*^- ^cells. (B) Southern blot analysis of *StuI *and *BciVI *digested genomic DNA to confirm the recombination event in *gxcDD*^- ^cells. Two independent clones were analysed. (C) Western blot analysis indicates the complete loss of GxcDD protein in *gxcDD*^- ^cells (upper panel). The blot was reprobed with actin specific mAb act1-7 as a loading control (lower panel).

To gain knowledge about the physiological role of GxcDD we disrupted the gxcDD gene by homologous recombination in wild type AX2 cells (Fig. 6A). The disruption of the gene was confirmed by PCR analysis using genomic DNA of individual clones and verified by Southern blot analysis and by western blotting of total cell lysates using polyclonal antibodies (Fig. 6B–C).

*gxcDD*^- ^cells did not show severely altered phenotypes when examined for growth on a bacterial lawn or in suspension, cytokinesis or actin organization in the cortex (not shown). The enrichment of the ArfGAP-PH domain at macropinosomes (Fig. 4A, B) suggested that GxcDD might have a role in endosomal processes like phagocytosis and pinocytosis. Quantitative phagocytosis and pinocytosis assays did however not reveal significant differences with the parental strain (not shown).

### *gxcDD*^- ^cells show a delay in development and defects in streaming behaviour

**Figure 7:**
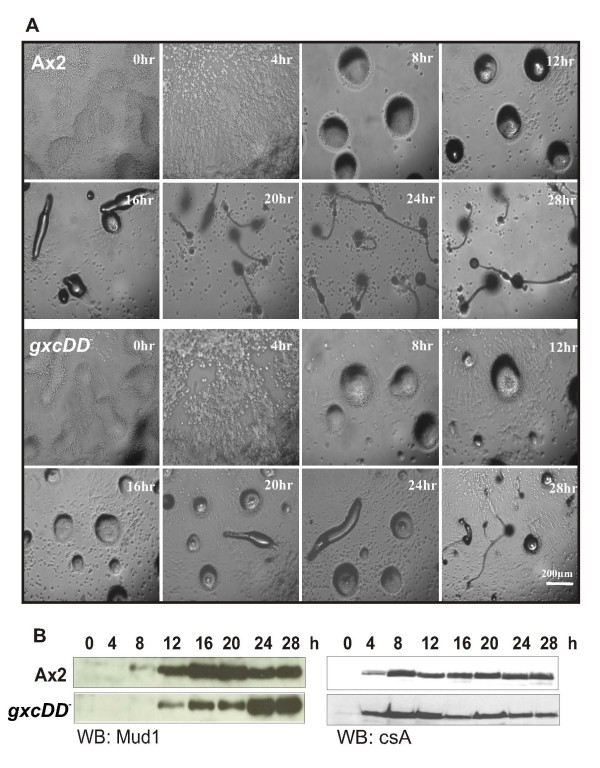
Developmental delay in *gxcDD*^- ^cells. (A) Parental wild-type AX2 and *gxcDD*^- ^cells were allowed to develop on nutrient deficient agar plates. Images were recorded every 4 hrs. *gxcDD*^- ^cells did develop to form fruiting bodies but were delayed in development by at least 4 hrs. (B) Cells were collected at 4 hr intervals for total protein and analysed by western blot. The appearance of the prespore marker pspA (detected by Mud1 monoclonal antibody) and the cell adhesion molecule csA (detected using monoclonal antibody 33-294-17) were monitored.

*D. discoideum *is a soil living organism feeding on bacteria; upon sensing starvation a developmental programme is initiated as a survival strategy. cAMP serves as the master regulator for development. In this process unicellular amoebae aggregate to form a mound, and the mound forms a tipped aggregate or an optional slug stage which shows phototactic and thermotactic behaviour. The developmental programme is completed upon reaching culmination and formation of the fruiting body. The entire process is well coordinated and programmed and wild type AX2 cells complete development by 20–24 hrs upon starvation when allowed to develop on nutrient deficient phosphate agar plates. *gxcDD*^- ^cells enter the developmental programme timely when starved and complete the developmental process by forming fruiting bodies. However the developmental programme is delayed, and fruiting bodies are formed only after 28 hrs (Fig. 7A). A closer analysis showed that the early developmental stages took place timely until cells had formed aggregates, however from t12 onward development was delayed by more than 4 hours. Tipped aggregates had formed only after 20 hours and slugs at t24 as compared to t16 for AX2. Moreover, not all aggregates had transformed into fruiting bodies at t28. This delay in development was also confirmed by analysing the expression profile of the prespore specific protein pspA which is recognized by mAb Mud1. In AX2 highest levels of pspA are observed between t12 and t20, whereas in *gxcDD*^- ^cells pspA reached similar levels from t20 to t28. By contrast, the aggregation specific cell adhesion protein contact site A (csA) had its peak expression at t8 in wild type and mutant (Fig. 7B) confirming the defect in later developmental stages.

**Figure 8:**
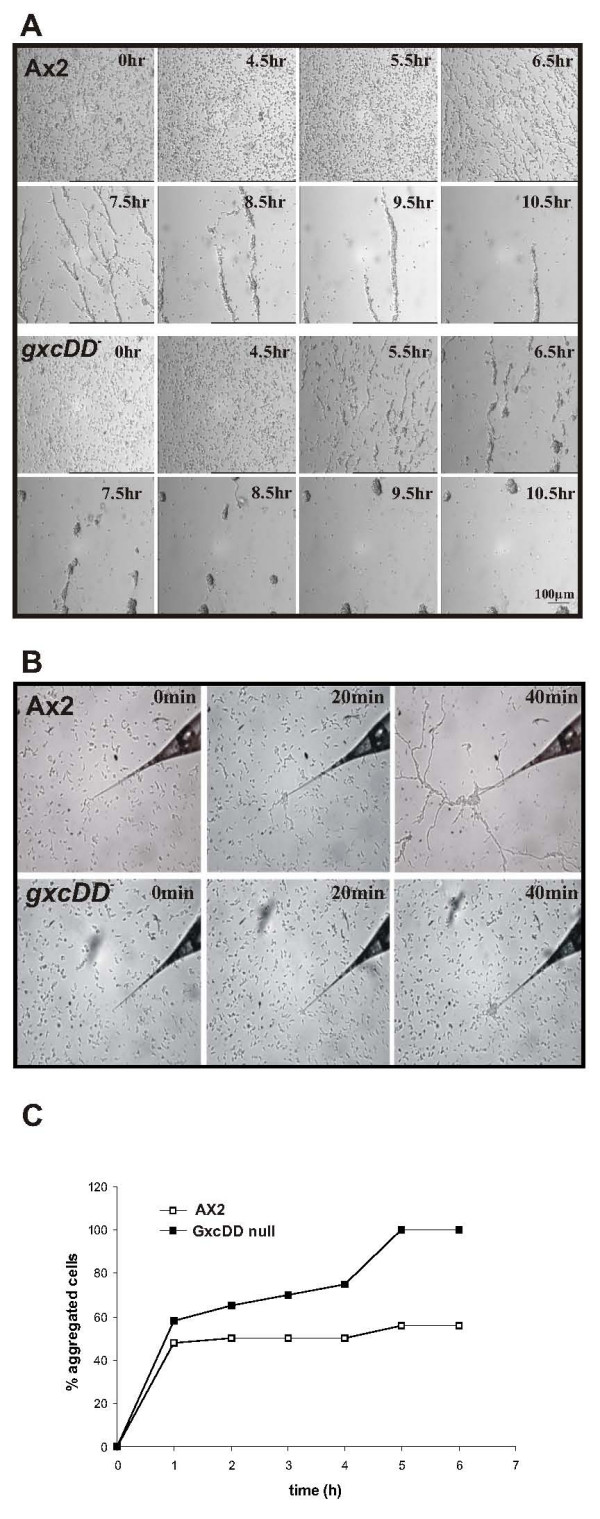
Streaming defect in *gxcDD*^- ^cells. (A) AX2 and *gxcDD*^- ^cells were allowed to starve on plastic plates. Images were continuously recorded. Streams formed in *gxcDD*^- ^cells broke down to aggregates. (B) Streaming was also recorded in wild type and *gxcDD*^- ^cells using an exogenous source of cAMP in a capillary chemotaxis assay. *gxcDD*^- ^cells did migrate towards the cAMP source but did not form streams. (C) *gxcDD*^- ^and AX2 cells were allowed to starve in Soerensen buffer at a density of 1 × 10^7 ^cells/ml and samples were withdrawn at the indicated times. The percentage of aggregated cells was determined by measuring the OD_600_. The data represent the average of three independent experiments.

When wild type AX2 cells are starved in a monolayer, cells polarize and form long streams. *gxcDD*^- ^cells were not able to form such long streams. Cells polarized and began to form streams, but soon the streams broke apart and were left behind as aggregates (Fig. 8A). Streaming of aggregation competent *D. discoideum *cells can also be observed in a capillary chemotaxis assay by applying an exogenous source of cAMP. *gxcDD*^- ^cells migrated towards the cAMP source but did not form characteristic streams as in wild type cells (Fig. 8B). We then assayed aggregate formation in suspension, which can be determined by following the decrease in optical density of the suspension due to aggregate formation. We found that the degree of aggregation was reduced in *gxcDD*^- ^cells (Fig. 8C) and a large majority of cells stayed as single cells and did not form aggregates.

**Figure 9:**
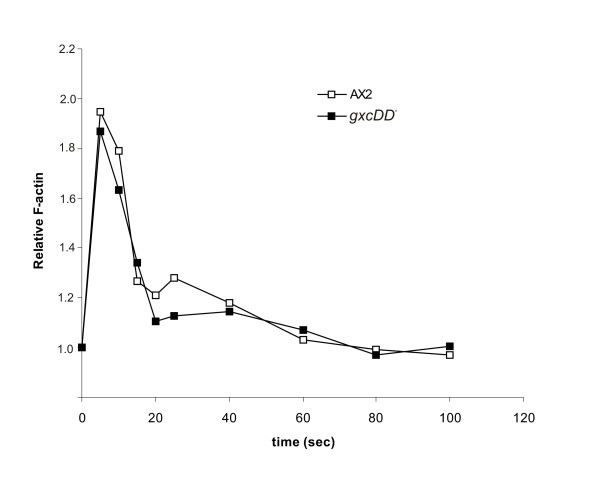
cAMP induced actin polymerization response in mutant and wild type cells. Aggregation competent cells at 2 × 10^7^cells/ml were stimulated with 1 μM cAMP and samples were taken at the indicated times. The F-actin content was determined as described in Materials and methods. The data is the average of three independent experiments.

A quantitative analysis of chemotaxis parameters in chemotaxing *gxcDD*^- ^cells showed that speed, persistence and directionality were similar to the AX2 wild type cells (Table 1). In agreement with these results quantitative measurement of F-actin levels upon cAMP stimulation was also similar to AX2 (Fig. 9A). Our observations of the subtle defects in development and streaming behaviour in *gxcDD*^- ^cells indicate that, although GxcDD is dispensable for development, it acts at various stages of development.

**Table 1:** Analysis of cell motility in *gxcDD*^- ^gells.

	AX2	gxcDD^-^
Buffer		
Speed (μm/min)	6.65 ± 2.61	5.53 ± 1.89
Persistance (μm/min-deg)	1.71 ± 1.19	1.38 ± 0.59
Directionality	0.31 ± 0.19	0.32 ± 0.18
Direction change (deg)	47.81 ± 11.94	49.06 ± 14.28
		
cAMP Gradient		
Speed (μm/min)	14.56 ± 3.81	15.03 ± 3.75
Persistance (μm/min-deg)	5.55 ± 2.25	5.52 ± 2.27
Directionality	0.71 ± 0.26	0.83 ± 0.14
Direction change (deg)	24.49 ± 17.76	17.32 ± 8.93

Time lapse image series were captured at 30 s intervals. The DIAS software was used to trace individual cells along the image series and calculate motility parameters.

## Discussion

In the present study we have analysed GxcDD, a novel putative *Dictyostelium *RacGEF that has a unique domain organization containing a CH domain, two IQ motifs, a RhoGEF domain followed by two PH domains and an ArfGAP domain followed by one more PH domain. Seven other *Dictyostelium *RacGEFs contain a CH domain [4]. The Calponin homology (CH) domain is a protein module of about 100 amino acids present in the actin-binding protein calponin that controls smooth muscle contraction and cytoskeletal organization in non-muscle cells. The CH domain of calponin is however not responsible for its interaction with actin [22]. In contrast, the actin-binding domain (ABD) of several proteins is comprised of two CH domains in a tandem arrangement. CH domains are classified into at least five classes based on the degree of sequence similarity [22,23]. CH1 in association with CH2 can interact with actin. Isolated CH2 and CH3 domains do not interact with actin at all [24,25]. CH4 and CH5 are found in the actopaxin/parvin family of actin-binding proteins implicated in linking integrins with intracellular pathways that regulate the actin cytoskeleton [26]. Most RhoGEFs from *Dictyostelium *and other species like mammalian Vav and α-PIX contain one type 3 CH domain and have been implicated in signal transduction. The CH domain of GxcDD is also a type 3 CH domain and our studies indicate that this domain is targeted to membranes exclusively and does not interact with actin. The membrane targeting of the CH domain may regulate the association of GxcDD with membranous fractions. CH3 domain containing proteins are the group of CH domain containing proteins that have the most diverse functions among the CH domains. Our findings are consistent with the hypothesis that although the five CH domains are homologous and have structural similarity, they may have evolved to perform different functions [27].

The DH domain of RhoGEFs regulates the nucleotide exchange activity. The associated PH domain is required for its full catalytic activity and for phosphoinositide binding and localization of the protein. Recent studies in yeast have shown that only a small fraction of PH domains are capable of independent membrane targeting of a protein and those that do often require phosphoinositides and non-phosphoinositide determinants like Arf GTPases in some cases for subcellular localization [28]. GxcDD possesses a DH domain which is followed by two PH domains, the first being comparatively large and poorly conserved. We found that the DH domain can bind to a set of Rac GTPases (Rac1a, RacA, RacC, RacE, RacH and RacI) with sufficient affinity. However, our data need to be handled with caution, as mere physical interaction does not imply that all the above-mentioned Racs would be substrates for GxcDD *in vivo*. Further characterization of the interaction by enzymatic exchange assays needs to be performed for deeper understanding of its GEF activity. It is also possible that some of the interacting Racs and GxcDD might never see each other *in vivo *because of their subcellular localization e.g., RacH is mostly located at endosomes and not at the plasma membrane [13].

A conformational change or binding of bioactive lipids to proteins are mechanisms known to activate a signal transducing protein [29,30]. Our observation of the ArfGAP-PH tandem binding to GxcDD but not being able to form higher oligomers indicates that the ArfGAP-PH may interact with some other part of the protein and exist as an inactive form. A conformational change would then be required to convert it to its active form. A possible activator could be PtdIns(3,4,5)P which binds to the ArfGAP-PH tandem with significant affinity. In an ArfGAP, ASAP1, a member of the centaurin family, which has an ArfGAP-PH tandem like GxcDD, the PH domain is known to function as an autoinhibitory domain and upon activation by PtdIns the ArfGAP domain is activated [31]. A similar activation mechanism may also exist for GxcDD. The recruitment of the ArfGAP-PH tandem to the cortical regions of the cell and the phagocytic cup indicates a possible role in endocytic processes.

To understand the physiological role of GxcDD, we generated *gxcDD*^- ^cells. Our observations indicate that although cells lacking GxcDD grow normally under laboratory conditions, undergo development and complete it with the formation of fruiting bodies, streaming behavior during chemotaxis is altered and development delayed. *Dictyostelium *cells can form streams during the aggregation process, which is the result of a cAMP relay mechanism. In this process certain cells, the pacemaker cells, have the ability to produce cAMP. The secreted cAMP can bind to cARs (cAMP receptors, which are G-protein coupled receptors), which induce a multitude of signaling events. Activation of cARs leads to dissociation of the heterotrimeric G-proteins and membrane localization of CRAC (*c*ytosolic *r*egulator of *a*denylyl *c*yclase), a PH domain containing protein, followed by activation of adenylyl cyclase (ACA), cAMP production and secretion [32]. Activation of ACA is also regulated by another cytosolic regulator, Pianissimo [33]. The neighboring cell then senses cAMP and streams of cells moving towards the aggregation centre are generated. A concerted action of 3-phosphoinositide metabolizing enzymes PI3K and PTEN are involved in tightly regulating the translocation of CRAC, Akt/PKB, PhdA (PH domain containg proteins) to the leading edge. Ras GTPases can activate PI3K. Ras C and its activator aimless RasGEF show decreased ACA activation. Cells lacking proteins that regulate ACA activity show defects in streaming or total loss and inability to aggregate or progress through development. It has been found that ACA localized to the rear of a migrating cell is required for formation of streams [34]. ACA is possibly localized to the rear by a vesicular system that would require Arf function and GxcDD might be a part in its regulation. As GcxDD contains several functional and regulatory domains, it is likely to regulate signal transduction downstream of cAMP receptors (cARs). Binding of cAMP to the surface ligand activates PI3K whose products may use GxcDD as adaptor protein to bring about downstream signalling. GxcDD at the membrane may have either a function in conjunction with GTPases with its GEF and GAP domain or may act along with another combination of functional domains. Interference with such a complex network of intricate signalling is a possible mechanism for the delay in developmental timing in *gxcDD*^- ^cells.

## Conclusion

GxcDD is an unusual RacGEF containing CH domain, two IQ motifs, a RacGEF-PH tandem and an ArfGAP-PH tandem. It is present in cytosolic as well as membranous fractions. The CH domain of GxcDD localizes to the plasma membrane and may recruit GxcDD to the membrane and possibly regulate function and localization of the protein by binding to the ArfGAP-PH tandem. This is to the best of our knowledge the first evidence of membrane targeting of a CH, which would reveal an additional function for this functionally diverse domain. Our studies show that the RacGEF domain has the ability to physically interact with several Rac GTPases. To get a better insight to GxcDD function we need to perform nucleotide exchange assays to find the actual substrates for GxcDD. The ArfGAP-PH domain is associated with the leading edge of the cell and is able to associate with phosphatidyl inositides. It is also present in phagosomes and in Triton X-100 insoluble fractions. Deletion of the gene did not result in major phenotypic changes associated with growth, endocytic processes, actin organization, cytokinesis and cell motility, but shows subtle defects in formation of streams during aggregation and a delay in development.

## Methods

### Strain growth and development

*D. discoideum *cells of strain AX2 were grown either with *Klebsiella aerogenes *on SM agar plates or axenically in liquid nutrient medium [35] in shaking suspension at 160 rpm at 21°C. *gxcDD*^- ^cells were cultivated in axenic medium containing 3.5 μg/ml blasticidin (ICN Biochemicals, OH). To analyse development, cells were grown axenically to a density of 2–3 × 10^6^/ml, washed twice in Soerensen phosphate buffer (17 mM Na-K phosphate, pH 6.0) and 5 × 10^7 ^cells were plated on phosphate agar plates. The streaming pattern was studied by allowing 1 ml of 5 × 10^6 ^cells/ml to settle on a well of a NUNC six-well plate and observed at 3 min intervals with a Leica DM-IL inverse microscope.

### Generation of *gxcDD*^- ^cells and molecular cloning

For disruption of the *GxcDD *gene in AX2 cells, a *GxcDD *gene replacement vector was constructed using the plasmid pBSBsrΔBam [36]. A 1.1-kb 5' fragment coding for the CH domain and the IQ motifs was PCR amplified using the forward primer 5-ATGCAACCCAAAGATTATATG-3'  and reverse primer 5-ACTATTGTAATGGATGAT-3' and a 1.0-kb 3' fragment was PCR amplified using forward primer 5'-TTAATGAGTTGTATGAGAAGA-3' and reverse primer 5'-TGTGCAGAATGTGGAGCATCA-3' from AX2 genomic DNA. The PCR products obtained were cloned into pGEM-T Easy cloning vector (Promega GmbH). The gene fragments were released and cloned into pBSBsrΔBam. The resulting replacement vector was linearised by digesting with PvuII and transformed into AX2 by electroporation. Transformants were selected in nutrient medium containing blasticidin (3.5 μg/ml). Independent clones were screened for the disruption of the GxcDD gene by PCR using genomic DNA, Southern blotting and western blot analysis. For Southern blot analysis a probe encompassing nucleotides 3807–4860 was used.

For expression of the CH domain of GxcDD fused to green fluorescent protein (GFP) at the N-terminus, a 0.4-kb fragment encoding the first 134 residues of GxcDD was amplified from AX2 cDNA and cloned into pBsr-GFP [37,38]. A 1-kb fragment encoding residues 395–707 containing the RacGEF domain was amplified and cloned in pGEX-4T1 (Amersham Biosciences) for expression in *E. coli*. The C-terminal 1-kb fragment encoding residues 1269–1619 containing the ArfGAP-PH tandem was amplified and cloned into pBsr-GFP for expression in AX2 cells and into pGEX-4T3 for expression in *E. coli*. Generation of strains expressing RacF1, RacG, RacH fused to GFP has been described elsewhere [12,13,39]. For expression of Rac1a, RacA (GTPase domain), RacB, RacC, RacD, RacE, RacI and RacJ fused to the C-terminus of GFP, PCR was performed on corresponding cDNAs. PCR products were cloned into pDEX-GFP [37] and the sequence verified. These vectors were introduced into AX2 cells and clones were selected by visual inspection under a fluorescent microscope.

### Generation of polyclonal antibodies specific for GxcDD

Polyclonal antibodies specific for GxcDD were obtained by immunising female white New Zealand rabbits with GST-ArfGAP-PH (100 μg/animal; Pineda Antikörper-Service, Berlin), followed by two boosts of 100 μg each at two weeks intervals. The antiserum was affinity purified by incubating with GST-ArfGAP-PH bound glutathione-sepharose beads.

### Subcellular fractionation

For separating membrane and cytosolic fractions cells were washed in Soerensen buffer and resuspended at a density of 1 × 10^8 ^cells/ml in MES buffer (20 mM MES, pH 6.5, 1 mM EDTA, 250 mM sucrose supplemented with protease inhibitor cocktail (Sigma)). Cells were lysed by sonication and membrane and cytosolic fractions separated by centrifugation at 100,000 *g *for 30 min at 4°C.

Triton X-100 was used for preparing cytoskeletal fractions. Cells were washed as before and resuspended in Soerensen buffer at a density of 5 × 10^7 ^cells/ml and 300 μl of cell suspension were lysed using an equal volume of TIC buffer (2% Triton X-100, 20 mM KCl, 20 mM imidazol, pH 7.0, 20 mM EGTA, 4 mM NaN_3_) and incubated on ice for 10 min followed by incubation at RT for 10 min. The insoluble cytoskeleton fraction was separated by centrifugation at 10,000 *g *for 4 min. Supernatant and pellet fractions were subjected to western blot analysis.

### GST pulldown assays

GST-RacGEF and GST-ArfGAP-PH were expressed in *E. coli *and bound to glutathione-sepharose beads. For interaction of GxcDD with Rac proteins, 4 × 10^7 ^AX2 cells expressing *Dictyostelium *Racs as GFP fusions were lysed in lysis buffer (25 mM Tris, pH 7.5, 150 mM NaCl, 5 mM EDTA, 0.5% Triton X-100, 1 mM NaF, 0.5 mM Na_3_VO_4_, 1 mM DTT, supplemented with protease inhibitors (Sigma)) and incubated with equal amounts of GST-RacGEF bound beads for 3 hrs at 4°C. Beads were washed with wash buffer (25 mM Tris, pH 7.5, 150 mM NaCl, 5 mM EDTA). The eluate of the pulldown was immunoblotted and the Rac protein detected using a GFP specific monoclonal antibody [40]. Cells expressing only GFP were used as a control. For interaction studies of the ArfGAP-PH domain GST-ArfGAP-PH bound to glutathione-sepharose beads was incubated with AX2 cell lysates. For interaction studies of the CH domain with RacGEF and ArfGAP-PH domain, glutathione-sepharose beads bound to GST fused RacGEF and ArfGAP-PH tandem were incubated with AX2 cells expressing GFP-CH domain. Pulldown eluates were immunoblotted and probed with GFP specific monoclonal antibody. GST bound beads were used as a control.

### Miscellaneous methods

Immunoflorescence was done by fixing cells using cold methanol for 10 min followed by staining for actin using actin-specific monoclonal antibody Act1-7 [41] and Cy3 conjugated secondary antibody. Live cell imaging of fluorescent cells in suspension or during phagocytosis of TRITC labelled yeast particles was done by laser scanning confocal microscopy essentially as described [42]. Capillary chemotaxis was done as described using a Leica DM-IL inverse microscope and analyzed using DIAS [43].

Monoclonal antibodies recognizing α-actinin [44], contact site A [45], pspA [46], comitin [47] and GFP [40] were used for western blotting.

F-actin levels upon cAMP stimulation were determined as described [48]. Briefly, aggregation competent cells resuspended at 2 × 10^7 ^cells/ml were stimulated with 1 μM cAMP and 50 μl samples were taken at various time points. Samples were immediately lysed in lysis buffer (3.7% formaldehyde, 0.1% Triton X-100, 0.25 μM TRITC-phalloidin in 20 mM potassium phosphate, 10 mM PIPES, pH 6.8, 5 mM EGTA, 2 mM MgCl_2_) and stained for 1 hr and centrifuged at 10,000 *g *for 5 min. Pellets were extracted with 1 ml methanol overnight and fluorescence (540/565) measured in a fluorimeter.

For crosslinking experiments GST-ArfGAP-PH was thrombin cleaved on glutathione-sepharose beads and the purified protein subjected to dialysis against PBS, pH 7.4, for 6 hrs at 4°C. To equal amount of protein increasing amount of glutaraldehyde (0–0.1% v/v) was added. The final reaction volume was 40 μl. Crosslinking was carried out at 4°C for 30 min. The reaction was stopped by addition of 5 μl 1 M glycine. Samples were subjected to imunoblotting.

Phosphoinositides binding was performed by a dot blot assay. 200 pmoles of each phophoinositide (PtdIns(3,4)P_2_, PtdIns(4,5)P_2_, PtdIns(3,4,5)P_3_) were spotted on a PVDF membrane and incubated with ArfGAP-PH domain. Protein bound to lipids was detected using polyclonal GxcDD antibodies.

For cell aggregation in suspension *gxcDD*^- ^and AX2 cells were allowed to starve in Soerensen buffer at a density of 1 × 10^7 ^cells/ml and samples were withdrawn at the indicated times. The percentage of aggregated cells was determined by measuring the OD_600_.

For northern blot analysis total RNA was isolated from AX2 cells of different developmental stages as described previously and separated in agarose gels containing 6% formaldehyde [49,50]. The blot was probed with a fragment derived from the 3' end of the GxcDD cDNA (nt 3807–4860)

## Authors' contributions

SM and DN were involved in carrying out all the experiments. SM drafted the manuscript. FR and AAN supervised the work and revised the manuscript.
